# Polymeric nanocapsular baicalin: Chemometric optimization, physicochemical characterization and mechanistic anticancer approaches on breast cancer cell lines

**DOI:** 10.1038/s41598-019-47586-7

**Published:** 2019-07-30

**Authors:** Riham I. El-Gogary, Sara A. Abdel Gaber, Maha Nasr

**Affiliations:** 1 Department of Pharmaceutics and Industrial pharmacy, Faculty of Pharmacy, Ain Shams University, Cairo, Egypt; 2 Nanomedicine Department, Institute of Nanoscience and Nanotechnology, Kafrelsheikh University, Kafrelsheikh, Egypt

**Keywords:** Targeted therapies, Breast cancer

## Abstract

Baicalin is a multi-purpose flavonoid known for its anticancer properties, but its application is hindered by its low water solubility and bioavailability. Polymeric nanocapsules were proposed in this work as a promising system for enhancing baicalin delivery, and potentiating its anticancer properties. The characterization of nanocapsules was augmented with chemometric analysis, and the selected formulations were tested on two breast cancer cell lines (MCF-7 and MDA-MB-231), with mechanistic anticancer elucidation using MTT assay, confocal microscopy uptake, flow cytometry, mechanism of cell death, reactive oxygen species production, caspase 3/7 activity and death biomarker expression using quantitative real time PCR. Results showed that baicalin nanocapsules displayed favorable pharmaceutical properties; with the formulation variables affecting their properties elucidated using chemometric factorial analysis. Nanocapsules enhanced the anticancer activity of baicalin up to 216 times for MCF-7 cells and 31 times for MDA-MB-231 after 24 hr incubation. Cellular internalization of the fluorescently labeled nanocapsules was confirmed after 4 hr incubation for both cell lines. Apoptosis was the dominant cell death mechanism, with significant up-regulation of P53 in baicalin nanocapsules treated cells. Data here presented drive to further preclinical studies to investigate the delivery of baicalin polymeric nanocapsules and their anti-cancer activity.

## Introduction

Currently, there is an increased need to explore nutraceuticals as potential anticancer agents, in order to overcome the pitfalls of conventional cancer therapies^1^. Among the promising nutraceutical molecules that has recently emerged is baicalin, which is a key flavonoidal compound commonly used in Chinese medicine, known for its strong anticancer activity^2,3^. Its anticancer properties are mainly attributed to its antiproliferative potential^4^, and its ability to inhibit the mobility of cancer cells^1^. Baicalin proved its prominent antitumor activity against various cancer types such as colon^5^, hepatic^6^, bladder^7^ and glioblastoma^8^. Many studies have also evaluated the cytotoxic effect of baicalin against breast cancer cells either *in vitro* or *in vivo*^9–11^. The particular efficacy of baicalin against breast cancer cells is the main focus of the current manuscript owing to the alarming statistical incidence of breast cancer worldwide^12^.

Despite its antitumor potential, baicalin suffers from low bioavailability (almost 2%) owing to its poor water solubility^13^. In order to enhance its bioavailability and effectiveness in cancer treatment, baicalin was formulated in different nanoparticulate systems, such as liposomes^3,13^ and solid lipid nanoparticles^2^. Nanoparticles are known to preferentially accumulate within tumors *via* the EPR effect^14,15^, and can be easily uptaken by the cells *via* endocytosis. In a recent study, gold nanoparticles were conjugated with baicalin and the conjugate induced a more prominent apoptosis in breast cancer cell line MCF-7^16^. However, metallic nanoparticles are not biodegradable and are known for their long-term toxic effects^17^. In this regard, the use of biocompatible and biodegradable polymers is favored.

A promising biodegradable nanoparticulate system which was proven effective in cancer treatment is polymeric nanocapsules^18–20^. Polymeric nanocapsules are composed of a polymeric coat and a lipophilic oily core, stabilized with hydrophilic and lipophilic surfactants^21^. Their advantages include the possibility of high drug loading into the oil core, their stable nature, and their ability to protect drugs against enzymatic degradation because of the presence of a polymer shell. Polymeric nanocapsules could reduce systemic toxicity and improve the pharmacokinetics of drugs. Polylactide-glycolide (PLGA) is a commonly used polymer for the preparation of nanocapsules, owing to its biodegradability, as well as its ability to be tailored for active targeting by covalent attachment of targeting ligands to its acid terminated functional groups.

Despite its lipophilicity, no attempts have been made to encapsulate baicalin in polymeric nanocapsules till current date. Moreover, only very few papers attempted to study the molecular mechanisms behind the anticancer activity of polymeric nanocapsules. Therefore, the aim of the current manuscript was to successfully prepare and optimize PLGA polymeric nanocapsules loaded with baicalin intended for administration *via* the IV route, with the influence of formulation variables on the key properties of nanocapsules being studied using chemometric full factorial design analyses. Another aim was to fully elucidate their cellular mechanistic behavior by studying their anticancer efficacy on two different breast cancer cell lines, their cellular uptake using confocal microscopy. Quantitative assessment of nanocapsules cellular accumulation was assessed using flow cytometry, with concomitant quantification of cellular apoptosis/necrosis, reactive oxygen species production and caspase 3/7 activity. Moreover, quantitative real time polymerase chain reaction was also conducted for death biomarkers expression.

## Materials and Methods

### Materials

Baicalin was purchased from Skin actives company, USA. Soybean phosphatidylcholine (Epikuron E145V) was kindly provided by Cargill company, Germany. Labrafil M2125 CS oil was kindly supplied by Gattefosse’ company, France. Polylactide-glycolide (PLGA) grade 7502 A (Purasorb PDLG of lactide:glycolide molar ratio 75:25 and inherent viscosity mid point 0.2 dl/g) was kindly provided by Purac company, Netherlands. Poloxamer P407 was kindly provided by BASF company, Germany. Potassium dihydrogen phosphate, Tween 80, glacial acetic acid, disodium hydrogen phosphate, ethanol, acetic acid, hydrochloric acid and acetone were purchased from El-Nasr pharmaceutical company, Egypt. Methanol and water HPLC grade were purchased from Fisher Scientific company, UK. Nanosep centrifugal tubes (M.wt 100 kD) were purchased from Pall company, Germany. Dialysis membrane (12–14000 Mwt cut off), dimethyl sulfoxide, Trolox and fluorescein isothiocyanate (FITC) were purchased from Sigma Aldrich, USA. 3-(4,5-dimethylthiazol-2-yl)-2,5 diphenyltetrazolium bromide (MTT) was purchased from Bio basic, Canada. The cell culture media RPMI1640, DMEM, penicillin-streptomycin mixture and biotase were purchased from Gibco, USA. Fetal bovine serum, trypsin and sterile phosphate buffer saline were purchased from Biochrom AG, Germany. All cell culture-related consumables were purchased from Corning, USA. Human breast carcinoma cell lines (MCF-7 and MDA-MB-231) were obtained from the Institut für angewandte Zellkultur, Germany.

### Preparation of baicalin loaded PLGA nanocapsules

Baicalin loaded nanocapsules were formulated using nanoprecipitation technique^18,22,23^, according to the factorial design attributes described in Table 1. Four mL of acetone and six mL of methanol (in which 10 mg baicalin, 0.5 gram Labrafil M2125 CS oil, 50 mg PLGA and 50 mg Epikuron E145V were dissolved) were introduced to 20 mL aqueous phase containing surfactant (either Tween 80 or Poloxamer P407) drop-wise using a syringe, and were stirred at room temperature till complete evaporation of the organic solvent mixture (Yellow line MAG HS7, IKA-Werke GMBH and Co., France). The formulations were kept at temperature 4–8 °C for further analysis.

**Table 1:** Factors and levels employed for the 2^3^ factorial design.

	Factor	Minimum Level	Maximum Level
X_A_	Type of hydrophilic surfactant	Tween 80	Poloxamer 407
X_B_	Concentration of hydrophilic surfactant	0.2%	0.4%
X_C_	Concentration of lipophilic surfactant (Epikuron E145V)	0.5%	1%

### Chemometric analysis of the particle size, polydispersity index (PDI) and zeta potential of nanocapsules

A 2^3^ chemometric full factorial model design was created to delineate the principal effects and interaction modes of 3 chosen factors on the particle size; polydispersity index and zeta potential of nanocapsules; namely the type of hydrophilic surfactant (either Tween 80 or Poloxamer 407), its concentration (0.2% or 0.4%), in addition to the concentration of lipophilic surfactant Epikuron E145V (0.5% or 1%)^24^. Twenty four formulations (three formulations per run) of baicalin loaded nanocapsules were prepared, and the complete first order regression model described was:

$${\rm{Y}}={{\rm{\beta }}}_{{\rm{0}}}+{{\rm{\beta }}}_{{\rm{1}}}{{\rm{X}}}_{{\rm{A}}}+{{\rm{\beta }}}_{{\rm{2}}}{{\rm{X}}}_{{\rm{B}}}+{{\rm{\beta }}}_{{\rm{3}}}{{\rm{X}}}_{{\rm{C}}}+{{\rm{\beta }}}_{{\rm{4}}}{{\rm{X}}}_{{\rm{A}}}{{\rm{X}}}_{{\rm{B}}}+{{\rm{\beta }}}_{{\rm{5}}}{{\rm{X}}}_{{\rm{A}}}{{\rm{X}}}_{{\rm{C}}}+{{\rm{\beta }}}_{{\rm{6}}}{{\rm{X}}}_{{\rm{B}}}{{\rm{X}}}_{{\rm{C}}}+{{\rm{\beta }}}_{{\rm{7}}}{{\rm{X}}}_{{\rm{A}}}{{\rm{X}}}_{{\rm{B}}}{{\rm{X}}}_{{\rm{C}}}+{\rm{\varepsilon }}$$

in which Y is the dependent variable, β_i_ is the multiple regression coefficient delineating the main effects and the two/three factor interactions, X_A_, X_B_, and X_C_ are the studied factors of the design, and ɛ is the residual model error.

The particle size, PDI and zeta potential of baicalin nanocapsules were measured using the Zetasizer device (Nano ZS, Malvern, UK) after being diluted with deionized water 1:1000. Measurement temperature was set to 25 °C with detection angle 173° after equilibration for 2 minutes, and refractive index 1.33^18,25–27^.

### Measurement of the entrapment efficiency of nanocapsules

The free drug was separated from the encapsulated drug using centrifugal concentration^20^ at 6000 rpm for 30 minutes (Hermle cooling centrifuge, Germany) using Nanosep tubes. The amount of baicalin in the filtrate was calculated using HPLC (Agilent, USA), following a previously validated reverse phase method^28,29^. Briefly, the chromatographic conditions were C18 column (Hypersil, Thermo Scientific), mobile phase methanol:water containing 0.5% acetic acid 94:6, and a detection wavelength 279 nm. The encapsulated drug was computed as follows^30,31^:

$$\mathrm{EE} \% =\frac{{\rm{Total}}\,{\rm{drug}}-{\rm{free}}\,{\rm{drug}}}{{\rm{Total}}\,{\rm{drug}}}\times 100$$

### Assessment of shelf life stability of nanocapsules

The baicalin nanocapsules properties (particle size, PDI, zeta potential) were re-assessed after storage in closed vials at 4–8 °C for three months, to delineate their stability^32^.

### Transmission electron microscopy (TEM) of nanocapsules

The selected nanocapsules formulations were examined for their morphology after negative staining with 1% uranyl acetate^33^. Samples were placed on a carbon grid, followed by drying and staining. A final drying step was conducted before examination using TEM (JEM-100S, Japan).

### *In-vitro* release of baicalin from nanocapsules

The release of baicalin from the selected nanocapsules formulations was done using a dialysis based method^34–37^. One mL of baicalin nanocapsules formulation was placed in a glass cylinder of length 7 cm and radius 1.25 cm, tied from one end with dialysis membrane (Molecular weight cut off 12000–14000) and the other end to the shaft of USP dissolution tester (Hanson Co., USA), rotating at 100 rpm and 37 °C. The release medium was 200 mL phosphate buffer pH 6.5^29^, ensuring sink conditions for baicalin. Samples were withdrawn from the medium at 0.25, 0.5, 1, 2, 3, 4, 6, 8 hr, and assessed for the quantity of released baicalin using HPLC as previously described^28,29^. The release data was kinetically treated to elucidate whether the release mechanism followed zero, first or diffusion mode, by computing the values of the corresponding regression coefficients.

### Anti- breast cancer activity of nanocapsules

MCF-7 and MDA-MB-231 cells were cultured in RPMI1640 and DMEM media respectively. Both media were supplied with 10% fetal bovine serum and 100 U/ml penicillin, 100 µg/ml streptomycin, kept at 37 °C with 5% CO_2_ in a humidified atmosphere. All cell culture work was performed under sterile conditions, and both cell lines were used throughout the whole study. The MTT assay was chosen to assess the cytotoxicity of the selected baicalin nanocapsules^18,23^.

Cells were seeded in sterile, tissue culture treated, flat bottom 96 well plates at a density of 1 × 10^5^ cell/100 µL of the 10% fetal bovine serum containing medium placed in each well and incubated overnight. A stock solution of the free baicalin was prepared in growth-maintenance medium (containing only 2% fetal calf serum) and supplied with 1% sterile dimethyl sulfoxide to assure complete drug solubilization. A serial dilution using the respective maintenance medium was prepared (0–2000 µg/mL) for the free baicalin and for the selected formulations (0–250 µg/mL). Samples were sonicated for 15 minutes before use to assure homogenous distribution of the nanocapsules. The old medium was replaced with 200 µL pre-warmed drug containing medium supplied with 2% fetal bovine serum. Control groups were included, in which cells were incubated with drug-free medium containing 1% dimethyl sulfoxide. Cells were cultured for either 24 or 48 hr. The morphological features of the cells were monitored by imaging representative wells using camera connected to the inverted microscope (Leica DMIL, Germany) at magnification of 20×. The medium was afterwards withdrawn and cells were washed with pre-warmed sterile phosphate buffer saline. Cells were incubated for further 4 hr at 37 °C in MTT containing complete medium (20 µL of MTT solution prepared as 5 mg/mL phosphate buffer saline per 100 µL of complete growth medium). The formed formazan crystals were dissolved in 100 µL of acidified isopropanol/well prepared as 1.5% v/v using hydrochloric acid. Plates were shaken for 1 minute at 150 rpm/minute using an orbital shaker (Cole-Parmer, USA) and the absorbance was measured at 560 nm using a microplate reader (BMG LABTECH, Germany). Results were corrected for blank readings, and the viability was calculated as a percentage from the control untreated cells according to the following equation:

$${\rm{Viability}}\, \% =\frac{{\rm{Absorbance}}\,{\rm{of}}\,{\rm{treated}}\,{\rm{cells}}-{\rm{Absorbance}}\,{\rm{of}}\,{\rm{blank}}}{{\rm{Absorbance}}\,{\rm{of}}\,{\rm{control}}-{\rm{Absorbance}}\,{\rm{of}}\,{\rm{blank}}}$$

The half inhibitory concentrations (IC50) were calculated using GraphPad Prism (7) (San Diego, USA) with confidence interval identified as 95%.

In order to verify the safety of the selected nanocapsules formulations, the MTT assay was performed on fibroblast cell line cultured in Dulbecco’s Modified Eagle’s Medium (DMEM) high glucose medium supplemented with 10% heated fetal bovine serum, 1% of 2 mM L-glutamine, 50 IU/mL penicillin and 50 µg/mL streptomycin. Cells were seeded at a density of 1000 cell/well, and the procedure described above was repeated.

### Confocal fluorescence microscopic examination of nanocapsules

To prepare fluorescently labeled nanocapsules, the selected nanocapsules formulations were prepared using the same methodology for preparation of nanocapsules mentioned before, after incorporating FITC dye in the oil core. Solution of FITC in ethanol at concentration of 1 mole% was prepared and added to the internal phase. All the procedures were performed in the dark.

Cells were seeded over cover slips and incubated overnight for growth. They were incubated for 4 hr with FITC labeled selected nanocapsules formulations (25 µL/mL medium). The medium was gently removed and cells were washed once with pre-warmed PBS before being fixed using ice cold methanol, and examined by confocal fluorescence microscope (Zeiss LSM710, Jena, Germany) (excitation at 488 nm and emission at 521 nm). All acquisition parameters in terms of zoom, gain and offset were fixed for all samples. A control group of cells not incubated with the labeled nanocapsules was included.

### Quantification of nanocapsules cellular internalization

For quantification of cellular accumulation of the fluorescently labeled nanocapsules, cells were cultured in T-25 flasks overnight. They were incubated with FITC labeled nanocapsules formulations at 50 µL/mL for 4 hr in 2% fetal bovine serum containing medium in the dark. Cells were washed, enzymatically detached from the flasks using trypsin, and centrifuged for 5 minutes at 1500 rpm. The pellet was washed once in ice cold phosphate buffer saline before the fluorescence of 30,000 cells/sample was measured using FACS Calibur flow cytometer (BD Biosciences, Belgium). Control group (incubated with medium only) was also included to account for the autofluorescence. The excitation and emission wavelengths were 495 and 519 nm respectively. Flow cytometry data was analyzed using Flowing software 2.5.1 (Turku University, Finland).

### Elucidating the anticancer mechanism of baicalin polymeric nanocapsules by apoptosis/necrosis quantification

Cells were seeded in T-25 flasks and treated with IC50 conditions determined by the MTT assay after 24 hr of incubation with either free baicalin or the selected nanocapsules formulations. Control group (incubated with medium only) was also included as reference. At the end of the incubation period, they were dissociated from the plates using biotase and collected by 5 minutes centrifugation at 1200 rpm. Following the manufacturer’s instructions, cells were stained for 15 minutes at room temperature with Annexin V FITC and propidium iodide (PI) supplied within the kit (BD Pharmingen FITC Annexin V apoptosis detection). Cells were suspended in the provided binding buffer and the fluorescence signal of both PI and Annexin V FITC were simultaneously measured by flow cytometry. The excitation and emission wavelengths for PI were 493 and 636 nm respectively. For every sample, fluorescence of 10,000 cells was measured. Three measurement controls were included; (1) unstained cells, (2) cells stained with PI only, (3) cells stained with Annexin V FITC only. Data was analyzed with the same software mentioned before and the cumulative sum of both early and late apoptotic cells present in the right lower and upper quadrants of the dot plot were presented.

### Quantification of the produced reactive oxygen species (ROS)

Following the manufacturer’s instructions, cells were seeded in 96 well plates and 2′,7′–dichlorodihydrofluorescein diacetate (DFFH-DA) was added to each well at a final concentration 25 µM, and incubated at 37 °C for 45 minutes to allow cellular penetration of the dye. DCFDA-cellular reactive oxygen species detection assay was purchased from Mitosciences Abcam, Germany). Cells were then treated for 24 hr with IC50 of either free baicalin or the selected nanoformulations. A control group of untreated cells was included. Additionally, a positive control was included in which cells were treated for the same period with 50 µM Tert Butyl Hydrogen Peroxide (TBHP). The medium was then changed and the fluorescence of the produced 2′,7′ dichlororfluorescein (DCF) was measured (Ex: 495 nm and Em: 522 nm) using a microplate reader (BMG LABTECH, Germany) after subtracting the blank fluorescence. Results were expressed with respect to the ROS level from the positive control group. To verify how the produced ROS influences cell toxicity, cells were treated for 24 hr with IC50 condition of free baicalin co-incubated with ROS scavenger (1 mM Trolox) and the produced ROS in addition to cell viability were measured as previously described. To examine the cytotoxic effect of the added ROS scavenger, a control group in which cells were incubated with 1 mM Trolox only was included and the viability was measured using MTT assay as previously described.

### Caspase 3/7 activity measurement

In this experiment, only apoptosis was detected by assessing the hydrolytic activity of apoptosis-associated Caspase 3/7 using (Apo-ONE Homogenous Caspase 3/7 Assay, Promega) kit as instructed by the manufacturer. Cells were treated for 24 hr with IC50 conditions of either baicalin or the selected nanocapsules. A control group of untreated cells was included and the provided Caspase3/7 substrate was added to the medium (1:1 v/v). Plates were left for 1 hr at room temperature and the fluorescence of the produced rhodamine 110 was measured (Excitation at 495 nm and emission at 522 nm). Caspase3/7 activity was expressed as fold elevation compared to the control untreated group.

### Quantitative real time polymerase chain reaction

Cells were treated for 24 hr with IC50 conditions of either free baicalin or the selected nanocapsules. A control group of untreated cells was included. The total RNA was extracted using RNeasy Mini Kit (Qiagen, Germany) as instructed by the manufacturer. It was quantified and checked for its integrity using NanoDrop 2000/2000c (Thermo Scientific, USA). 1 µg was reverse transcript into cDNA using QuantiTect Reverse Transcription Kit (Qiagen, Germany) as instructed by the manufacturer. Quantitative RT-PCR was then conducted using SYBR green PCR kit (Qiagen, Germany) using StepOne Systems (Thermo scientific, USA) following the standard conditions. Sixty cycles of PCR were conducted as follows: 10 minutes at 95 °C (holding stage), 15 seconds for denaturation at 95 °C, 30 seconds for annealing at 60 °C and another 15 seconds for elongation at 60 °C. The primers used are shown in Supplementary 1. GADBH was used for normalization and the relative expression was calculated using 2^−ΔΔCT^ method.

### Statistical analysis

All experiments were done at least in triplicate. Statistical analysis was performed by Graphpad Instat software using ANOVA and Tukey post-test (P < 0.05). The factorial model analysis was performed using Design Expert software v.11.

## Results and Discussion

### Preparation of baicalin loaded PLGA nanocapsules

Baicalin nanocapsules were successfully prepared as a novel delivery system for the anticancer baicalin. The utilized polymer (PLGA) was commonly used in the preparation of nanocapsules delivering anticancer molecules^18–20^, owing to its biodegradable properties^38,39^. The oily core of the nanocapsules was constituted by a corn oil pegylated ester (Labrafil M2125 CS), providing the highest solubility for baicalin, based on a preliminary conducted solubility study (data not shown). In addition to nanocapsules, Labrafil M2125 CS was used as oil in the preparation of self microemulsifying delivery systems^40^. Epikuron was used as the lipophilic surfactant of choice, as it was reported to effectively stabilize the nanocapsules, in addition to its biodegradability and biocompatibility^18^. The hydrophilic non-ionic surfactants of choice were Tween 80 and Poloxamer 407, which were reported to enhance the anticancer activity of drugs or possess cytotoxic activity in tumor treatment^41–43^. The properties of the prepared nanocapsules are shown in Table 2.

**Table 2:** Characterization of baicalin nanocapsules.

Formula code	X_A_	X_B_	X_C_	Particle size (nm)	PDI	Zeta potential (mV)	EE%
F1	Tween 80	0.2%	0.5%	210 ± 2.90	0.225 ± 0.01	−47.3 ± 0.78	86.3 ± 1.23
F2			1%	201 ± 2.05	0.246 ± 0.03	−42.5 ± 4.38	90.8 ± 3.83
F3		0.4%	0.5%	189 ± 5.84	0.234 ± 0.03	−36.2 ± 2.69	85.1 ± 1.49
F4			1%	169 ± 3.00	0.214 ± 0.01	−36.9 ± 1.48	94 ± 2.05
F5	Poloxamer P407	0.2%	0.5%	215 ± 2.60	0.268 ± 0.02	−45.2 ± 0.85	87 ± 1.07
F6			1%	205 ± 2.95	0.229 ± 0.02	−30.0 ± 0.56	92.9 ± 0.91
F7		0.4%	0.5%	202 ± 0.60	0.374 ± 0.01	−34.1 ± 0.20	85.3 ± 3.01
F8			1%	179 ± 3.82	0.301 ± 0.03	−32.5 ± 3.39	93.9 ± 1.63

### Chemometric adequacy of the particle size, PDI and zeta potential factorial models

In order to investigate the influence of three independent surfactant-related variables (type and concentration of hydrophilic surfactant, and the concentration of lipophilic surfactant) on three dependent attributes of the nanocapsules (particle size, polydispersity and charge), a full factorial design was attempted.

The particle size of the nanocapsules ranged from 169–215 nm (Table 2), which is considered suitable for internalization within cancer cells^14^. As shown in Supplementary 2, the concentration of the hydrophilic surfactant was the major influencer on the particle size of nanocapsules, followed by the concentration of lipophilic surfactant then the type of hydrophilic surfactant, as can be inferred from their corresponding F values. As displayed in Supplementary 3, it was evident that increasing the concentrations of either hydrophilic or lipophilic surfactants resulted in a significant decrease in the particle size (P < 0.05). Their combined effect led to a dual decrease of the nanocapsules size, as could be inferred from the high F value of their corresponding two-factor interaction. The inverse relationship between particle size and concentration of surfactants is attributed to the surface tension lowering ability of the latter, resulting in better particle partitioning^44^. The use of Poloxamer resulted in nanocapsules of larger particle size than Tween 80 (P < 0.05), which might be ascribed to the higher adsorption potential of the latter surfactant^44^. The linear regression model as obtained from the factorial study was:

$${\rm{Particle}}\,{\rm{size}}=196.25+4{\rm{A}}-11.5{\rm{B}}-7.75{\rm{C}}+{\rm{1}}{\rm{.75AB}}-0.5{\rm{AC}}-3{\rm{BC}}-0.25{\rm{ABC}}$$

Statistical analysis of the model indicated its extreme significance (P < 0.0001), showing R^2^ value of 0.9671, adjusted R^2^ value of 0.9527 and a predictive R^2^ value of 0.926. The two latter terms were in reasonable agreement (less than 0.2), suggesting the adequacy of the model. The Box-Cox plot for power transformation generated by plotting the power against the natural log of the residual sum of squares delineated that the first order regression model was the best fit for the stated data (Supplementary 4).

On the other hand, the type of surfactant had the most prominent effect on the PDI of the nanocapsules, followed by the concentration of hydrophilic then lipophilic surfactants (Supplementary 5). Concurring with the results of the particle size, the increase in the particle size occurring upon using Poloxamer 407 rather than Tween 80 was accompanied by a significant increase in the PDI values of the nanocapsules (P < 0.05), suggesting the creation of more homogenous nanocapsules population with Tween 80. The effect of surfactant concentration on PDI was contraversial, in which the increase in hydrophilic surfactant concentration led to an overall significant increase in the PDI of the nanocapsules, while the opposite happened with the lipophilic surfactant (P < 0.05) (Supplementary 6). The linear regression model as obtained from the factorial study was:

$$\begin{array}{l}{\rm{PDI}}=0.2614+0.0316{\rm{A}}+0.0194{\rm{B}}-0.0139{\rm{C}}+0.0251{\rm{AB}}-0.0141{\rm{AC}}-0.0094{\rm{BC}}+0.0009{\rm{ABC}}\end{array}$$

Statistical analysis of the model indicated its extreme significance (P < 0.0001), showing R^2^ value of 0.8871, adjusted R^2^ value of 0.8377 and a predictive R^2^ value of 0.7459. The two latter terms were in reasonable agreement (less than 0.2), suggesting the adequacy of the model. The Box-Cox plot for power transformation generated by plotting the power against the natural log of the residual sum of squares also delineated that the first order regression model was the best fit for the stated data, and no transformation was needed (Supplementary 4).

Regarding the zeta potential of nanocapsules, both the type and concentration of surfactants had significant effects on the charge of the nanocapsules (P < 0.05). As shown in Supplementary 7, the concentration of hydrophilic sufactant had the most prominent effect on the zeta potential of nanocapsules, in which the increase of the concentration of the surfactant from 0.2% to 0.4% led to a significant decrease in nanocapsules charge (P < 0.05) (Supplementary 8). Similarly, the increase in concentration of lipophilic surfactant from 0.5% to 1% resulted in a significant decrease in the charge of nanocapsules (P < 0.05), but not as prominent as the hydrophilic surfactant concentration. The decrease of the charge on the surface of the nanocapsules upon increasing either hydrophilic or lipophilic surfactants could be attributed to their non ionic nature, which might shield the negative charges of the acid terminated PLGA polymer. As also shown in the figure, the use of Poloxamer resulted in nanocapsules of lower zeta potential values (P < 0.05) than when using Tween 80 as surfactant, which is probably attributed to their higher charge shielding properties^18^. The linear regression model as obtained from the factorial study was:

$$\begin{array}{l}{\rm{Zeta}}\,{\rm{potential}}=-\,38.09+2.64{\rm{A}}+3.16{\rm{B}}+2.61{\rm{C}}-1.01{\rm{AB}}+1.59{\rm{AC}}-2.39{\rm{BC}}\\ \,-1.01{\rm{ABC}}\end{array}$$

Statistical analysis of the model indicated its extreme significance (P < 0.0001), showing R^2^ value of 0.9072, adjusted R^2^ value of 0.8666 and a predictive R^2^ value of 0.7913. The two latter terms were in reasonable agreement (less than 0.2), suggesting the adequacy of the model. The Box-Cox plot for power transformation generated by plotting the power against the natural log of the residual sum of squares also delineated that the first order regression model was the best fit for the stated data, and no transformation was needed (Supplementary 4).

### Measurement of the entrapment efficiency of nanocapsules

As shown in Table 2, baicalin was successfully loaded in nanocapsules with high EE% values ranging from 85–94%, owing to its high solubility in the oily core and its lipophilic nature (log P 1.27)^29^. Upon further inspection of the results, only the concentration of lipophilic surfactant affected the EE% of the nanocapsules, in which the increase in the concentration of Epikuron resulted in a significant increase in the EE% of baicalin (P < 0.05), which might be attributed to the overall increase in lipophilicity of the nanocapsules by the incorporation of higher concentration of phospholipid.

### Assessment of the stability of nanocapsules

As shown in Supplementary 9, nanocapsules exhibited only minor changes in their particle size, PDI and zeta potential values upon storage. The high zeta potential values of nanocapsules can be considered the reason behind their stability, since they delay the nanocapsules aggregation through repulsive forces. The zeta potential values of the stored nanocapsules didn’t decrease below −30 mV, indicating physically stable nanocapsular dispersions even after storage^45^. A stability of at least 30 days was considered long term colloidal stability for nanocapsules, as delineated by other authors^46^.

### TEM of nanocapsules

**Figure 1:**
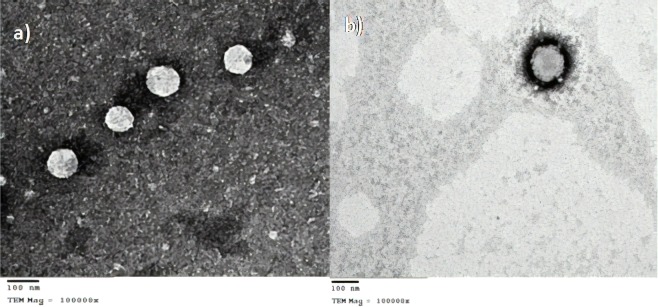
TEM micrographs of the selected formulation F4 (**a**) and F8 (**b**) at a magnification of 100000X.

Based on the aforementioned experiments, formulations F4 and F8 displaying the smallest particle sizes of the Tween 80 and Poloxamer nanocapsules respectively were selected for further characterization. As displayed in Fig. 1, both formulations displayed an obvious coat and core structure, with Poloxamer nanocapsules displaying a thicker coat than their Tween 80 counterparts, which concurred with the larger particle size of the former.

### *In-vitro* release profiles of nanocapsules

**Figure 2:**
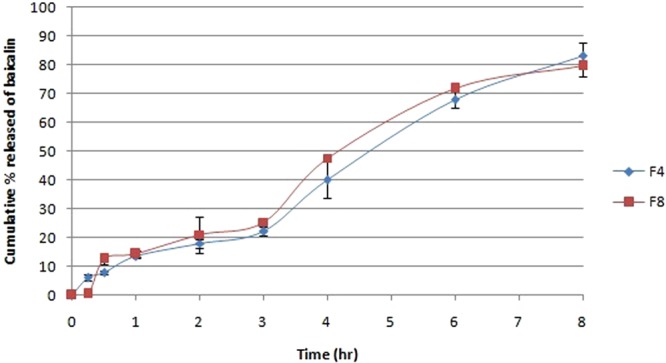
Cumulative percent released of baicalin from F4 and F8 over a period of 8 hr (n = 3).

The release profiles of formulations F4 and F8 were displayed in Fig. 2. As obvious from the figure, the formulations displayed similar release profiles up to a period of 8 hr. Upon kinetic fitting of the release data in the zero, first and diffusion equations, it was found that the release of baicalin followed zero order kinetics, as delineated by the highest regression coefficient value. This could be attributed to the reservoir-like morphology of the prepared nanocapsules, in which baicalin is contained within the oily care and is gradually diffusing across the polymeric membrane. The treatment of cancer benefits from the sustained and controlled release nature of the nanoparticles, since after their accumulation inside the tumor, they allow the sustained release of the drug over time at a constant rate, correlating with effective anticancer treatment.

### Anti- breast cancer activity of nanocapsules using MTT assay

Two commonly studied human breast carcinoma cell lines were used, namely MCF-7 and MDA-MB-231. They differ at the molecular level, where the latter cells are known to be triple negative breast cancer cells for their very low expression level of the breast carcinoma surface receptors; estrogen receptor (ER), progesterone receptor (PR), and human epithelial receptor 2 (HER2), whereas MCF-7 cells are known for their expression of both ER and PR^47^. Before conducting this experiment, the selected nanocapsules were tested in preliminary work to assess their safety on both cancer cells and normal cells. The blank nanocapsules (prepared without baicalin) were proven safe in MCF-7 cell line as delineated by the high viability percentages of cells at all tested concentrations. Moreover, both selected formulations F4 and F8 were tested on normal cells (fibroblasts), and they did not exhibit any cytotoxic effect as demonstrated in Supplementary 10, suggesting the selectivity of their action to cancer cells.

**Figure 3:**
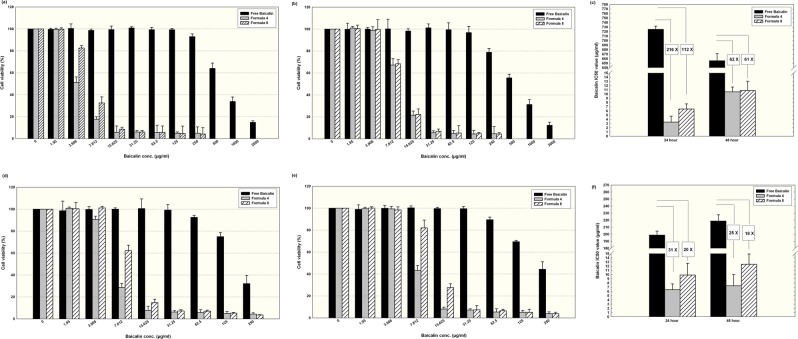
Viability results of human carcinoma cell line MCF-7 treated for 24 hr (**a**), 48 hr (**b**) and MDA-MB-231 cells treated for 24 hr (**d**) and 48 hr(**e**) using either free baicalin or loaded in PLGA nanocapsules F4 or F8 (Mean ± standard error, n = 5). (**c**,**f**) Show a comparison between the IC50 values of the free baicalin and the tested nanocapsules (F4 and F8) at both incubation periods (24 and 48 hr) for MCF-7 cells and MDA-MB-231, respectively.

As depicted in Fig. 3, for MCF-7 cells after 24 hr of incubation (7a), free baicalin concentration up to 250 µg/mL did not exert a significant reduction in cell viability compared to the control. Doubling the incubation period to become 48 hr (7b) slightly enhanced the exerted cytotoxic activity as illustrated by the reduction in the cell viability to almost 80%. On the other hand, using polymeric nanocapsules resulted in rapid significant reduction in cell viability, with formulation F4 exerting a more pronounced antineoplastic action than F8 after 24 hr of incubation for concentrations below 15.6 µg/mL (7a) (P < 0.05). This delineates the efficacy of Tween 80 as a hydrophilic surfactant to be used for nanocapsules preparation compared to Poloxamer 407 when it comes to cellular internalization within breast cancer cells, which may probably be attributed to the lower particle size of F4 compared to F8 (P < 0.05). However, both formulations were equi-potent when the incubation period was 48 hr (7b). Considering IC50 as a comparison parameter, baicalin concentration in F4 and F8 was 216 and 112 times lower than free baicalin when the incubation period was 24 hr (7c). After 48 hr, there was no statistically significant difference between the anticancer activity of both formulations, and the IC50 values were 61–62 times lower than the free baicalin.

Despite the resistance of MDA-MB-231 cells, they responded to free baicalin so that no further concentration than 250 µg/mL was required to reach IC50 at both tested incubation periods. Similar to the findings of MCF-7 cells, F4 exerted a significantly higher cytotoxic effect than F8 for concentrations below 15.6 µg/mL at incubation period of 24 hr (7d) and when the concentration was below 31.2 µg/mL at incubation period of 48 hr (7e). Similar to the findings obtained with MCF-7 cells, a reduction in polymeric nanoparticle efficiency was observed at 48 hr compared to 24 hr. For MDA-MB-231 being responsive to free baicalin, the polymeric nanocapsules lowered the concentration needed to reach IC50 conditions maximally 31 times compared to free baicalin (7f). The use of PLGA nanoparticles for the delivery of chemotherapeutic agents against breast cancer has been reported in the literature. PLGA nanoparticles of similar size and zeta potential were used for targeting doxorubicin to the nucleus and were tested *in vitro* on MDA-MB-231^48^. Moreover, in agreement with our findings, the drug loaded in nanoparticles showed a more potent anti-tumor action compared to the free drug^49^.

**Figure 4:**
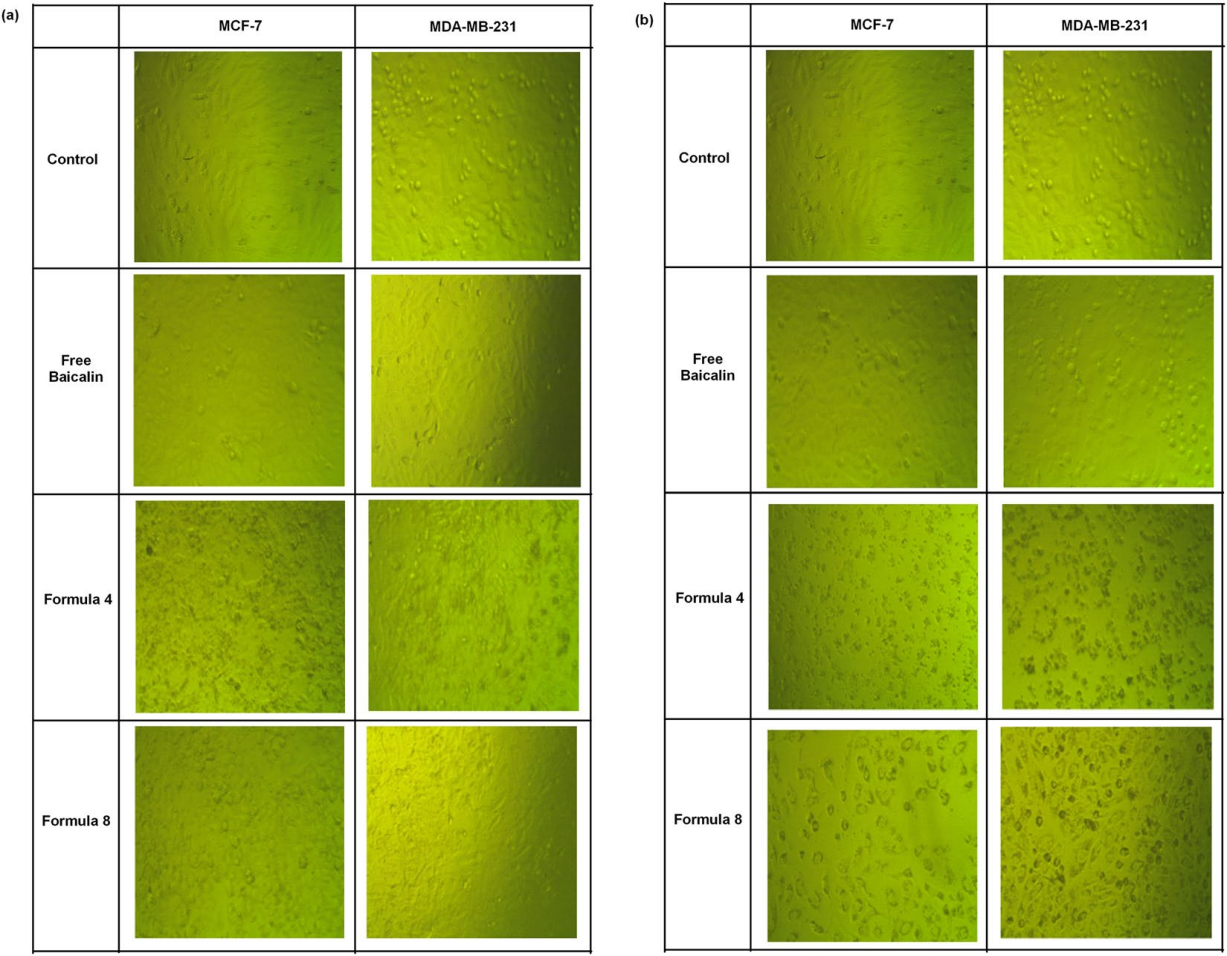
Morphological appearance of MCF-7 and MDA-MB-231 cells treated with (**a**) 7.8 µg/ml Baicalin and (**b**) 15.6 µg/ml for 24 hr either in the free form or encapsulated within F4 or F8 in contrast to a control of untreated cells.

Using phase contrast examination, morphological alterations of the cells were photographed. Figure 4 is a representation of both MCF-7 and MDA-MB-231 cells after treatment for 24 hr with (a) 7.8 µg/mL baicalin either in the free form or encapsulated within F4 or F8 and (b) 15.6 µg/mL of same drug forms. With respect to the control untreated cells, at concentration of 7.8 µg/mL, free baicalin did not impose any morphological alteration which is in agreement with the viability results for both cell lines shown in Fig. 3. On the other hand, MCF-7 cells lost their integrity and many granular bodies showing condensation of cellular materials appeared in the field in addition to floating cells when treated with F4. As for MDA-MB-231 cells, granulation appeared for some of the cells while the rest were still intact and adherent. When the cells were treated with the same concentration encapsulated within F8, the onset of cellular condensation formation was more obvious morphologically in MCF-7 cells in comparison to MDA-MB-231 ones. With the increase in the concentration, cells lost their characteristic monolayer appearance, the number of the granulation increased dramatically and the number of adherent cells decreased as illustrated in Fig. (4b). Cells treated with the free baicalin were morphologically similar to the control, which matches the viability results. The association between cytotoxicity and morphological alterations is in agreement with what was reported before^50^.

### Confocal fluorescence microscopy and flow cytometric assessment of nanocapsules

**Figure 5:**
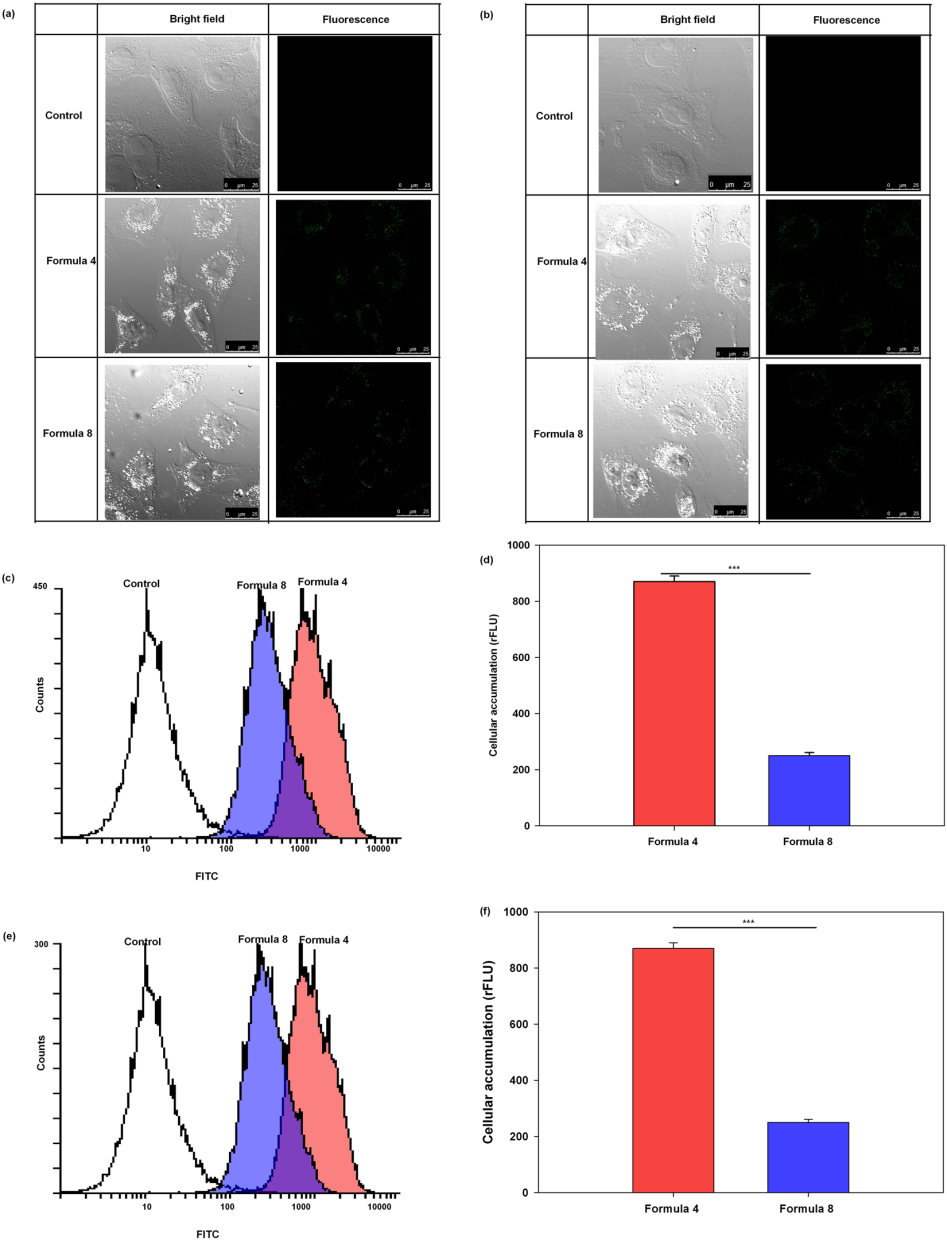
Confocal fluorescence representative images of (**a**) MCF-7 and (**b**) MDA-MB-231 cells incubated for 4 hr with either F4 or F8 labeled with FITC. For quantification, flow cytometry overlay histotograms of the (**c**) MCF-7 and (**e**) MDA-MB-231 cells incubated with either of the forms are depicted for 30,000 cell/sample. The measured relative fluorescence unit for (**d**) MCF-7 and (**f**) MDA-MB-231 cells are presented as bar charts (Mean ± standard error, n = 3).

Both nanocapsules formulations achieved adequate cellular localization after 4 hr of incubation as shown in Fig. 5. Both cell lines were free from significant autofluorescence as confirmed by the lack of fluorescence signals for the control groups. Both nanocapsules formulations were distributed within the cellular cytoplasm as shown in Fig. 5. The high cellular uptake of the nanocapsules was probably achieved by phagocytosis following strong non-specific interactions with the plasma membrane^51^. Flow cytometry analysis revealed that for both cell lines, F4 showed a significantly higher cellular accumulation that F8, further explaining the superiority of F4 over F8 in the viability experiment, which could also be ascribed to the smaller size of the former, in addition to the difference in surfactant composition.

### Delineating the mechanism of cellular death upon treatment with nanocapsules

**Figure 6:**
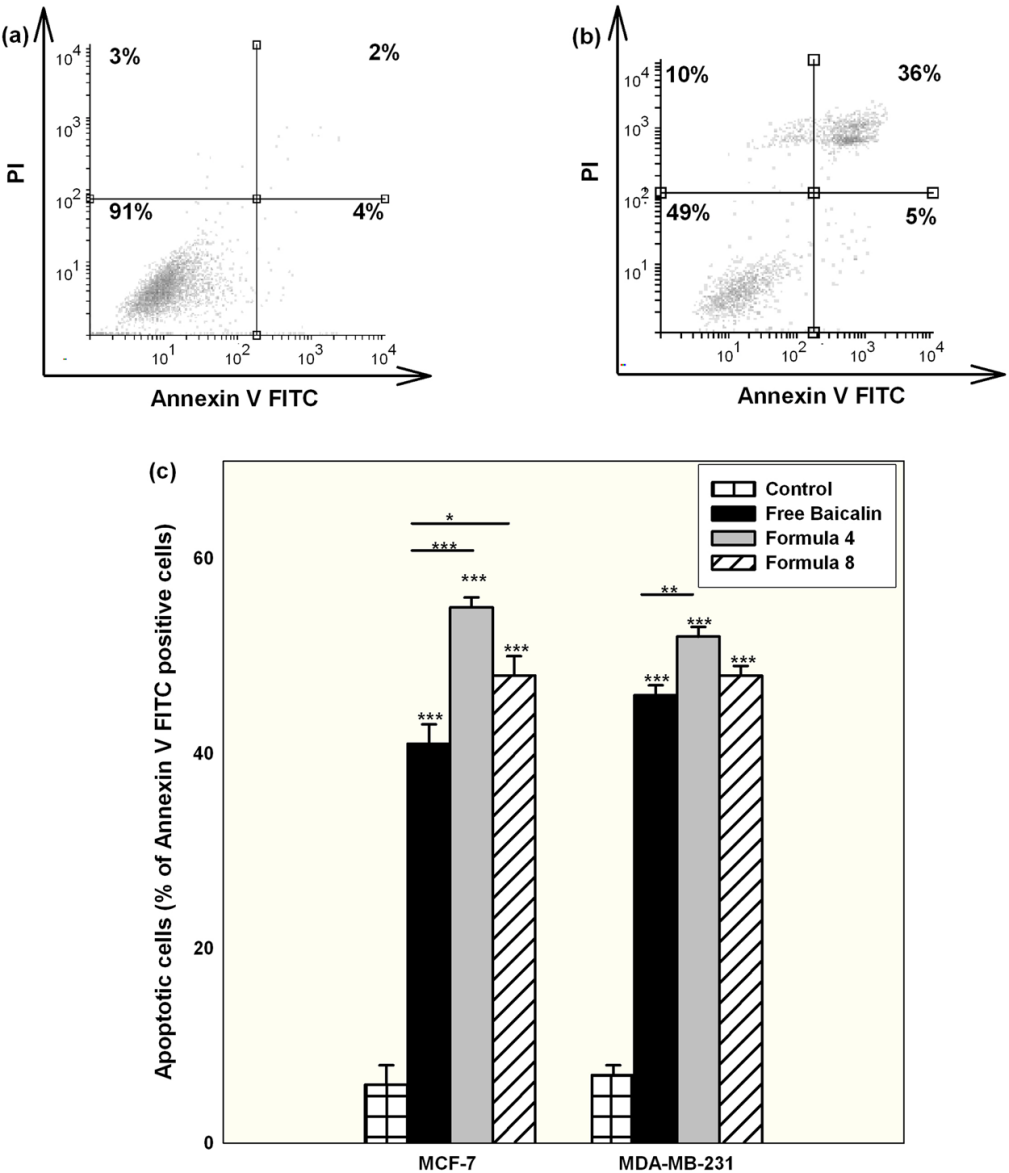
Representative histograms of MCF-7 cells stained with Annexin V FITC and PI for the detection of cell death mechanisms for (**a**) control untreated group and (**b**) treated with free baicalin at IC50 conditions for 24 hr. (**c**) Shows the percentage of the apoptotic cells (both early and late apoptosis) quantified for MCF-7 and MDA-MB-231 cells after being treated for 24 hr with IC50 conditions of either free baicalin or encapsulated within F4 or F8 and compared with the control untreated group (Mean ± standard error, n = 3, Statistical significance: P value *less than 0.05, **less than 0.01, ***less than 0.001).

Cells treated with free baicalin showed a predominant apoptotic cell death mechanism as indicated by a significant high cell population positive for Annexin V FITC (both early and late apoptosis) compared to the necrotic ones positive for PI. Using the polymeric nanocapsules of either formulations pronounced the apoptotic fraction of the cells significantly, with F4 causing the highest apoptotic cell fraction as illustrated in Fig. 6. Since F4 exhibited higher cellular accumulation than F8 as proven by the flow cytometry results, it was expected that F4 will elicit a better apoptotic effect.

Those findings concerning the mechanism of cell death were in agreement with previously reported ones, that baicalin exerts its antineoplastic action mainly through the induction of apoptosis irrespective of the cancer type used^5,52,53^. It is worth mentioning that baicalin apoptotic mechanism of cell death is not altered by its inclusion in nanoparticles, even when chemically conjugated to nanosystems^16^.

### The apoptotic cell death mechanism marked with an elevated oxidative stress, elevated caspase 3/7 activity, up-regulation of the pro-apoptotic P53 and bax and down-regulation of the anti-apoptotic Bcl-2

**Figure 7:**
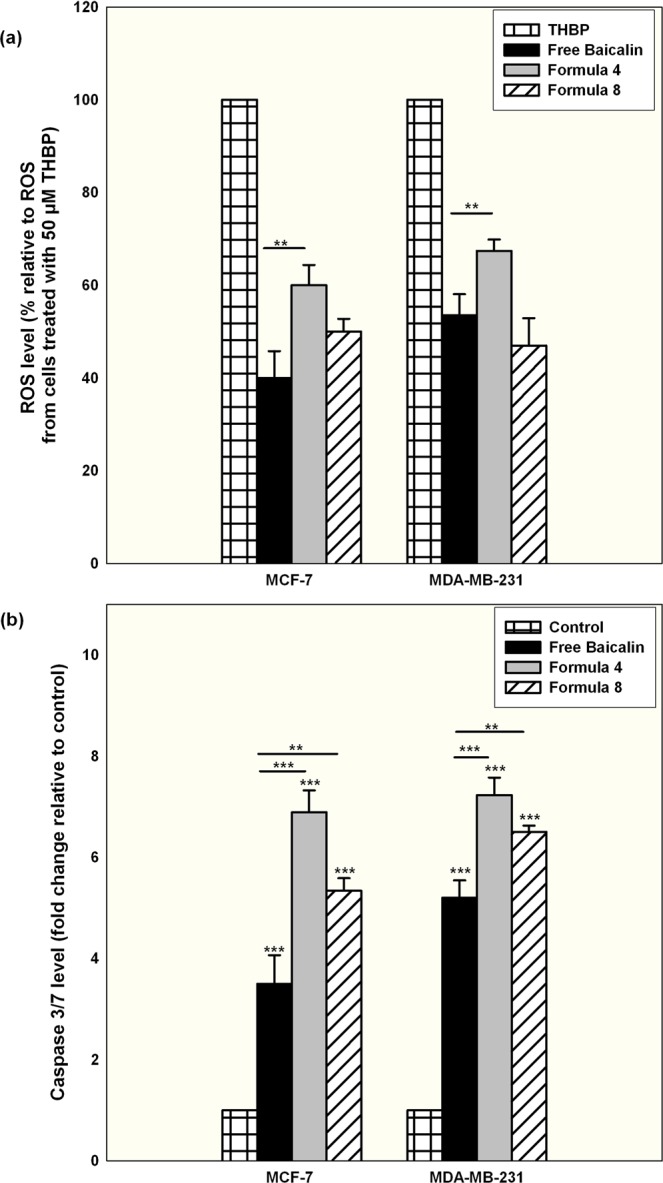
Quantification of ROS level of MCF-7 and MDA-MB-231 cells treated for 24 hr with IC50 conditions of baicalin either in the free form or encapsulated within F4 or F8 (**a**) with respect to the positive control of cells treated with 50 µM THBP. (**b**) Shows the quantification of Caspase 3/7 levels measured for MCF-7 and MDA-MB-231 cells treated for 24 hr with IC50 conditions of baicalin either in the free form or encapsulated within F4 or F8 expressed as fold change with relative to the control untreated cells (Mean ± standard error, n = 3, Statistical significance: P value *less than 0.05, **less than 0.01, ***less than 0.001).

**Figure 8:**
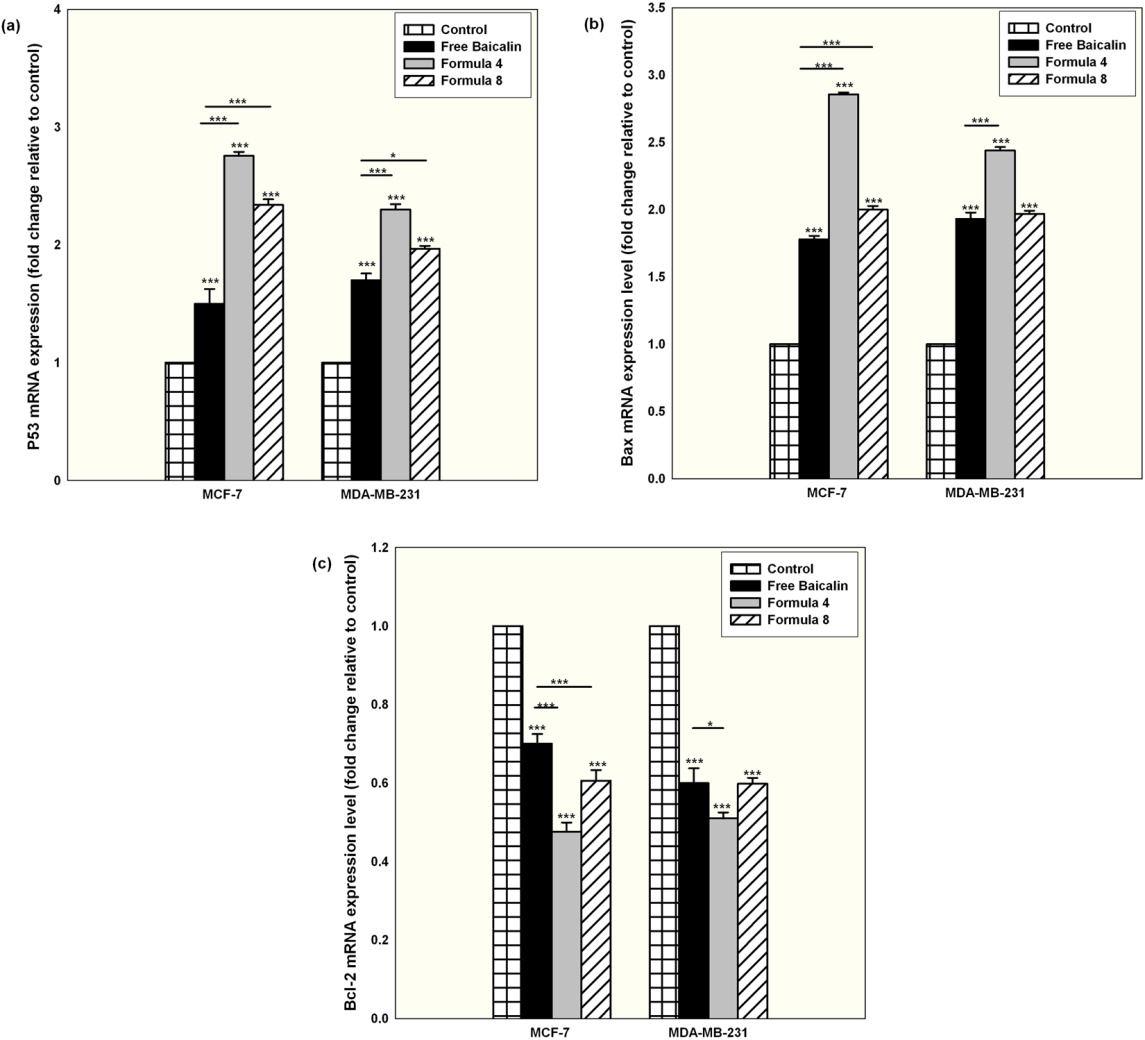
mRNA expression levels of (**a**) P53, (**b**) Bax and (**c**) Bcl-2 for both MCF-7 and MDA-MB-231 cells treated for 24 hr with IC50 conditions of free baicalin or encapsulated within formula 4 or 8. Presented results are normalized to the expression level of the house keeping GADPH and compared to the control untreated cells (Mean ± standard error, n = 3, Statistical significance: P value *less than 0.05, **less than 0.01, ***less than 0.001).

To investigate the signaling pathway for the exerted apoptosis, the level of intracellular ROS was measured for cells treated with either free or nanocapsular baicalin. As illustrated in Fig. (7a), with respect to the positive control (cells treated with 50 µM THBP), free baicalin treatment was associated with a significant elevation in ROS level for both tested cell lines (P < 0.05). Using the polymeric nanocapsules and in particular F4, a significant elevation in the level of intracellular ROS occurred. The produced ROS was totally quenched when ROS scavenger Trolox was co-incubated and at this condition, the associated cytotoxic effect was completely attenuated, proving the vital role played by ROS for the induction of cell death (Supplementary 11). The increase in ROS level coincided with an elevation of Caspase 3/7 in both cell lines and for all the tested forms as illustrated in Fig. (7b), with F4 showing the highest increase in Caspase 3/7 for both cell lines. Those findings are in accordance with the reported data about the ability of baicalin to cause an oxidative stress followed by an increase in the level of Caspase 3/7^50,54,55^. At the same time, mRNA expression level normalized to the expression level of the house keeping GADPH and compared to the normal control cells showed a significant elevation in the expression level of the pro-apoptotic P53 and Bax with a down-regulation of the anti-apoptotic Bcl-2 as shown in Fig. 8 for all tested conditions. It is worth mentioning that F4 displayed a more prominent effect for both cell lines at all tested mRNA expression levels. Up-regulation of P53 and Bax induced by baicalin treatment concurred with the results of other authors^56^. Those results further prove that the sustained baicalin release behavior mediated by PLGA nanocapsules was translated to an elevated level of markers involved in the intrinsic apoptotic mitochondrial dependant death signaling pathway. Moreover, they demonstrate the critical role that the cellular internalization of the nanocapsules plays in dictating their cytotoxic effect in cancer cells.

## Conclusions

PLGA nanocapsules displayed favorable physicochemical properties, and were proven promising in potentiating the anticancer activity of baicalin, owing to their better internalization within the cancer cells. Mechanistic studies as quantitiative real time PCR, assessment of Caspase 3/7 and elucidating the mechanism of cell death were proven valuable in delineating the anticancer mechanisms of baicalin nanocapsules by augmentation of mediated intrinsic apoptotic oxidative stress-based and mitochondrial dependant cell death mechanism. Data here presented drive to further preclinical studies to investigate the delivery of baicalin polymeric nanocapsules and their anti-cancer activity. Moreover, the optimized formulation obtained from the current work will be tailored for active targeting and further tested mechanistically in futuristic studies. Also in a general sense, this study opens opportunities for the delivery of other nutraceuticals whose applications are haltered by their physicochemical properties.

## Supplementary information

Supplementary file
