# Supplementary material for: Polymeric nanocapsular baicalin: Chemometric optimization, physicochemical characterization and mechanistic anticancer approaches on breast cancer cell lines
Source: Sci Rep. 2019 Jul 30;9:11064. doi: 10.1038/s41598-019-47586-7 (PMC6667692; doi:10.1038/s41598-019-47586-7)

**Polymeric nanocapsular baicalin: Chemometric optimization, physicochemical characterization and mechanistic anticancer approaches on breast cancer cell lines**

**Riham El-Gogary^a^, Sara A. Abdel Gaber^b^, Maha Nasr^a*^**

^a^Department of Pharmaceutics and Industrial pharmacy , Faculty of Pharmacy, Ain Shams University, Cairo, Egypt

^b^Nanomedicine Department, Institute of Nanoscience and Nanotechnology, Kafrelsheikh University, Kafrelsheikh, Egypt

**Supplementary 1: Primers used for PCR**

| Gene | Forward primer | Reverse primer |
| --- | --- | --- |
| P53 | 5′-GTGGAAGGAAATTTGCGTGT-3′ | 5′-CCAGTGTGATGATGGTGAGG-3′ |
| Bcl-2 | 5′- GGTGCCACCTGTGGTCCACCTG-3′ | 5′-CTTCACTTGTGGCCCAGATAGG-3′ |
| Bax | 5′-CAGCTCTGAGCAGATCATGAAGACA-3′ | 5′-GCCCATCTTCTTCCAGATGGTGAGC-3′ |
| GADBH | 5′-AGGGCTGCTTTTAACTCTGGT-3′ | 5′-CCCCACTTGATTTTGGAGGGA-3′ |

**Supplementary 2: Summary of the nanocapsular particle size chemometrics derived from the factorial design**

|  | **Sum of squares** | **df** | **Mean square** | **F value** | **P value** |
| --- | --- | --- | --- | --- | --- |
| Model | 5296.5 | 7 | 756.64 | 67.25 | <0.0001 |
| A | 384 | 1 | 384 | 34.13 | <0.0001 |
| B | 3174 | 1 | 3174 | 282.1 | <0.0001 |
| C | 1441.5 | 1 | 1441.5 | 128.12 | <0.0001 |
| AB | 73.5 | 1 | 73.5 | 6.53 | 0.0211 |
| AC | 6 | 1 | 6 | 0.533 | 0.4758 |
| BC | 216 | 1 | 216 | 19.2 | 0.0005 |
| ABC | 1.5 | 1 | 1.5 | 0.1333 | 0.7198 |

Supplementary 3: Particle size contour plots of baicalin nanocapsules (n=3)


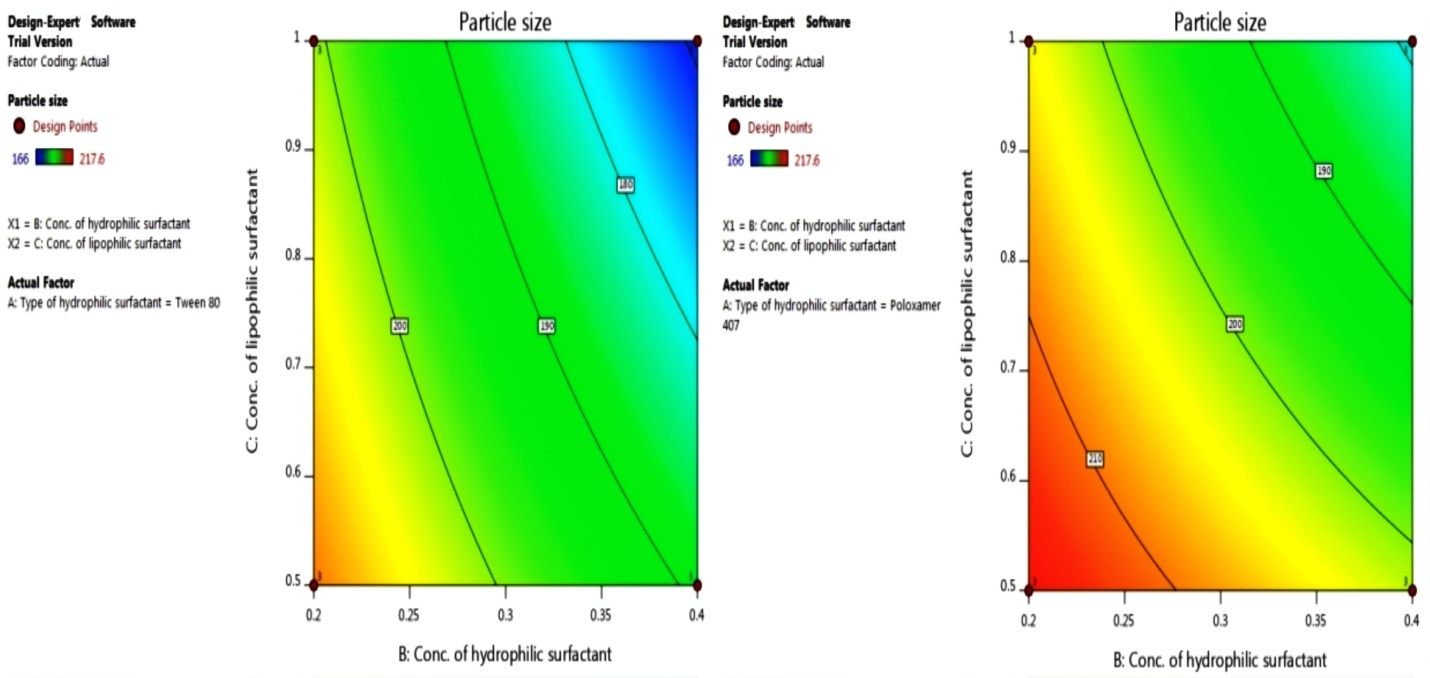


**Supplementary 4: Box Cox plots for power transformation for the particle size, PDI and zeta potential factorial models**

**
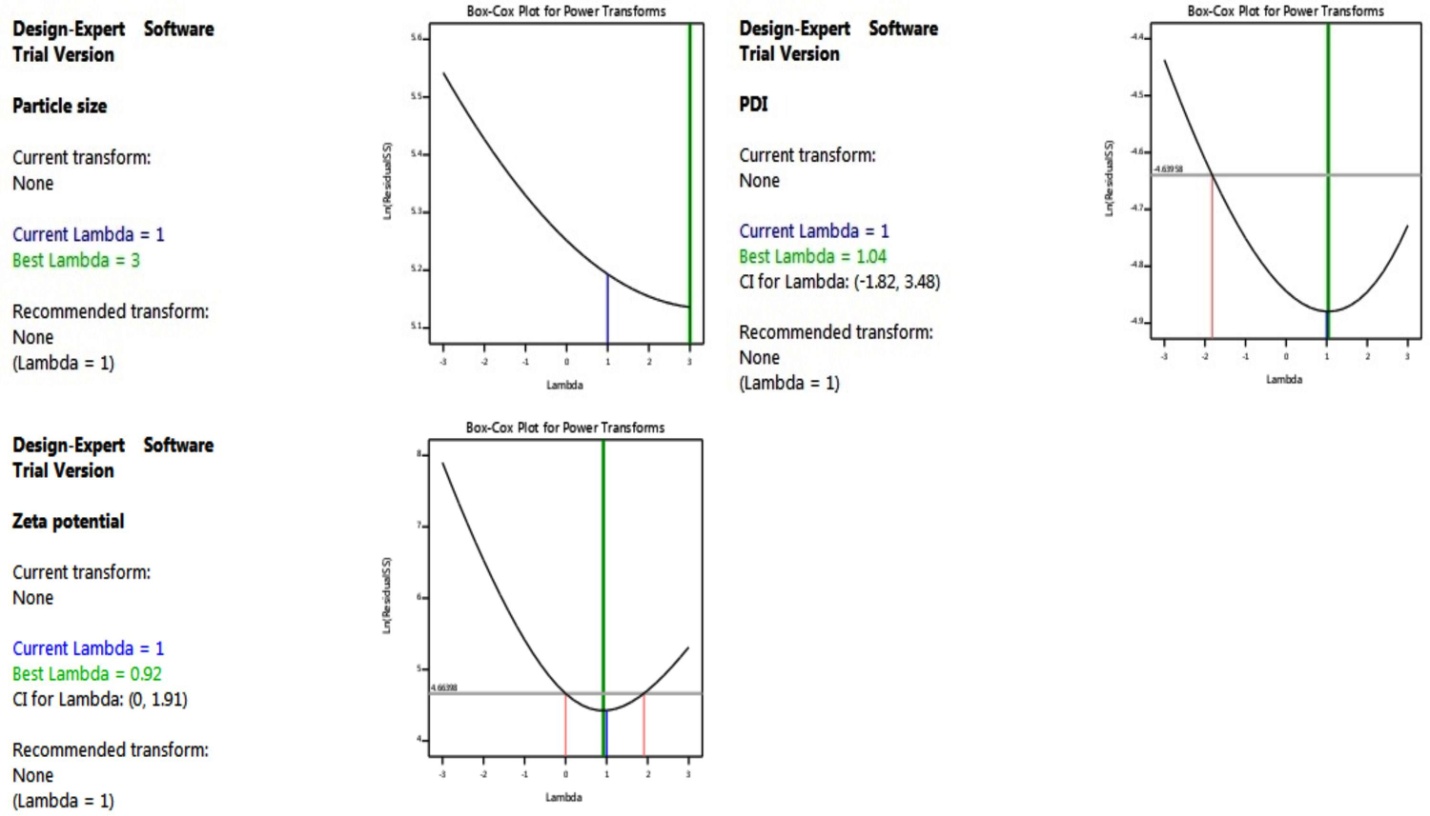
**

**Supplementary 5: Summary of the nanocapsular PDI chemometrics derived from the factorial design**

|  | **Sum of squares** | **df** | **Mean square** | **F value** | **P value** |
| --- | --- | --- | --- | --- | --- |
| Model | 0.0597 | 7 | 0.0085 | 17.95 | <0.0001 |
| A | 0.024 | 1 | 0.024 | 50.53 | <0.0001 |
| B | 0.009 | 1 | 0.009 | 18.97 | 0.0005 |
| C | 0.0046 | 1 | 0.0046 | 9.73 | 0.0066 |
| AB | 0.0152 | 1 | 0.0152 | 31.9 | <0.0001 |
| AC | 0.0048 | 1 | 0.0048 | 10.08 | 0.0059 |
| BC | 0.0021 | 1 | 0.0021 | 4.44 | 0.0512 |
| ABC | 0 | 1 | 0 | 0.0387 | 0.8466 |

Supplementary 6: PDI contour plots of baicalin nanocapsules (n=3)


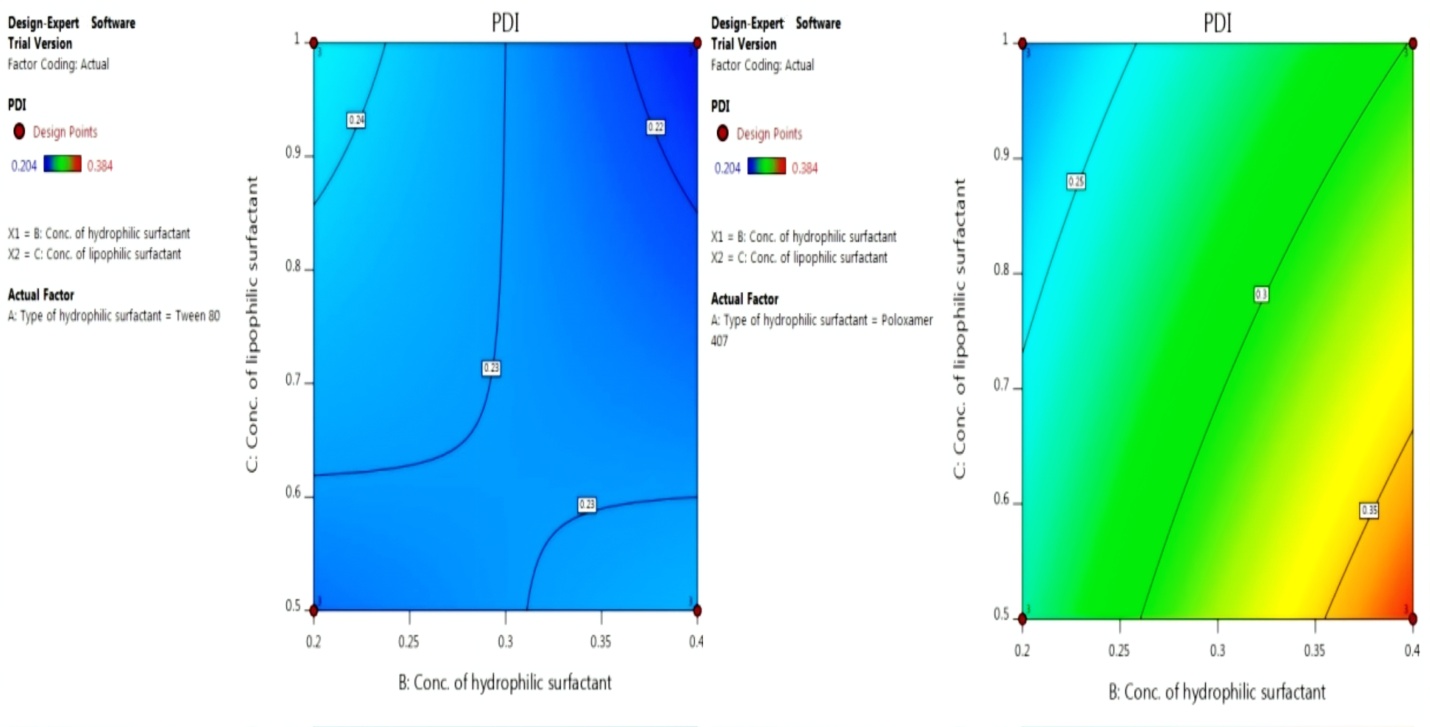


**Supplementary 7: Summary of the nanocapsular zeta potential chemometrics derived from the factorial design**

|  | **Sum of squares** | **df** | **Mean square** | **F value** | **P value** |
| --- | --- | --- | --- | --- | --- |
| Model | 817.29 | 7 | 116.76 | 22.35 | <0.0001 |
| A | 166.95 | 1 | 166.95 | 31.96 | <0.0001 |
| B | 240.03 | 1 | 240.03 | 45.95 | <0.0001 |
| C | 163.8 | 1 | 163.8 | 31.36 | <0.0001 |
| AB | 24.6 | 1 | 24.6 | 4.71 | 0.0454 |
| AC | 60.48 | 1 | 60.48 | 11.58 | 0.0036 |
| BC | 136.8 | 1 | 136.8 | 26.19 | 0.0001 |
| ABC | 24.6 | 1 | 24.6 | 4.71 | 0.0454 |

Supplementary 8: Zeta potential contour plots of baicalin nanocapsules (n=3)


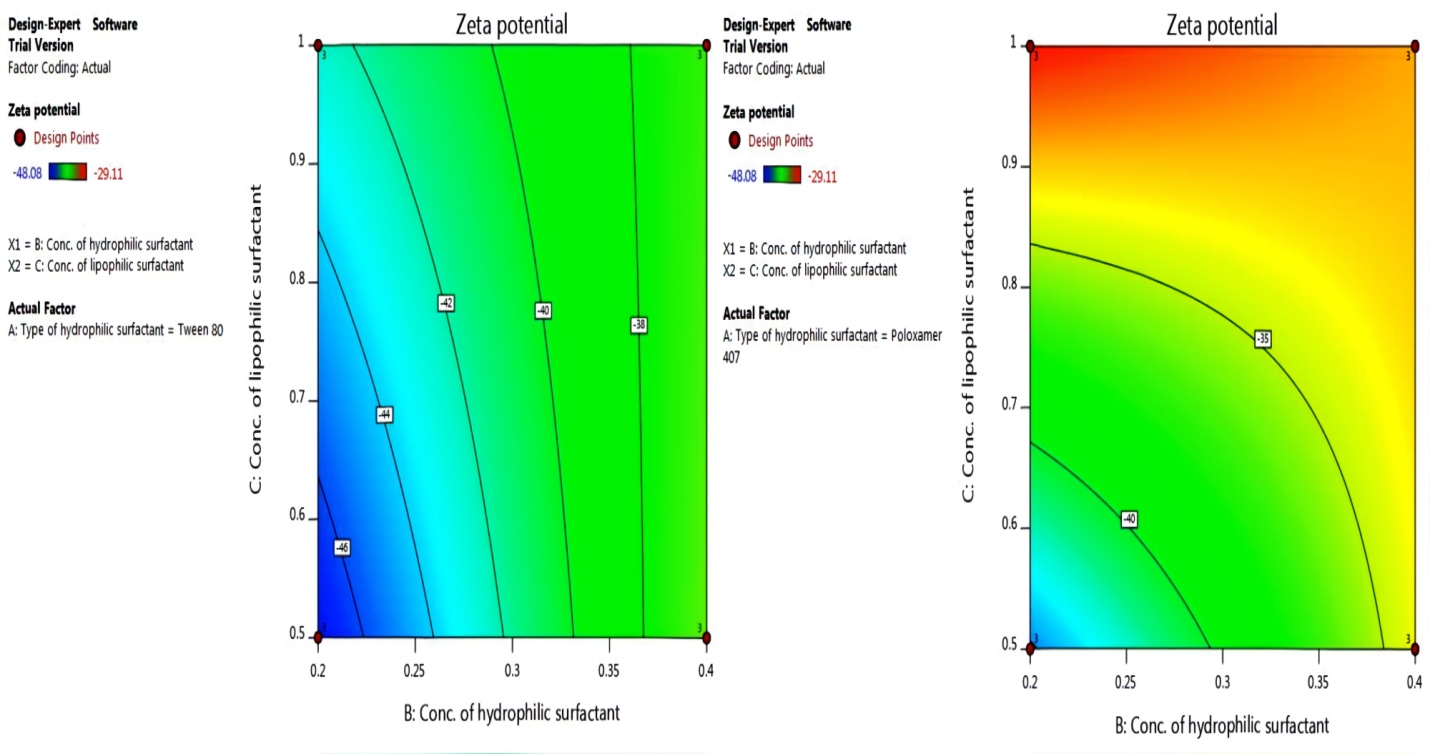


**Supplementary 9: Effect of storage on the particle size, PDI and zeta potential of nanocapsules**

| **Code** | **Particle size (nm)** | | **PDI** | | **Zeta potential**  **(mV)** | |
| --- | --- | --- | --- | --- | --- | --- |
|  | **Fresh** | **After storage** | **Fresh** | **After storage** | **Fresh** | **After storage** |
| F1 | 210±2.90 | 221±2.87 | 0.225±0.01 | 0.259±0.00 | -47.3±0.78 | -37.2±1.16 |
| F2 | 201±2.05 | 229±4.10 | 0.246±0.03 | 0.342±0.03 | -42.5±4.38 | -44.2±0.12 |
| F3 | 189±5.84 | 193±1.02 | 0.234±0.03 | 0.263±0.01 | -36.2±2.69 | -31.2±0.82 |
| F4 | 169±3.00 | 169±2.83 | 0.214±0.01 | 0.208±0.02 | -36.9±1.48 | -43.5±0.53 |
| F5 | 215±2.60 | 212±1.38 | 0.268±0.02 | 0.233±0.02 | -45.2±0.85 | -35.2±0.81 |
| F6 | 205±2.95 | 212±1.65 | 0.229±0.02 | 0.242±0.01 | -30.0±0.56 | -36.3±0.70 |
| F7 | 202±0.60 | 208±4.55 | 0.374±0.01 | 0.202±0.02 | -34.1±0.20 | -29.9±0.44 |
| F8 | 179±3.82 | 218±2.36 | 0.301±0.03 | 0.302±0.02 | -32.5±3.39 | -29.6±0.29 |

**Supplementary 10: Fibroblast cell viability percentages upon treatment with formulations F4 and F8**

**
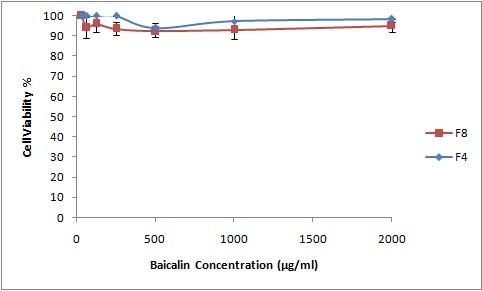
**

Supplementary 11: Verification of the importance of ROS level in exerted cell toxicity a)ROS level measured for MDA-MB-231 cells untreated or treated with either the positive control of THBP, or free baicalin at IC50 condition at 24 hour or baicalin and ROS scavenger trolox b) viability of MDA-MBA-231 cells either untreated or treated with either trolox or free baicalin IC50 value or free baicalin and trolox


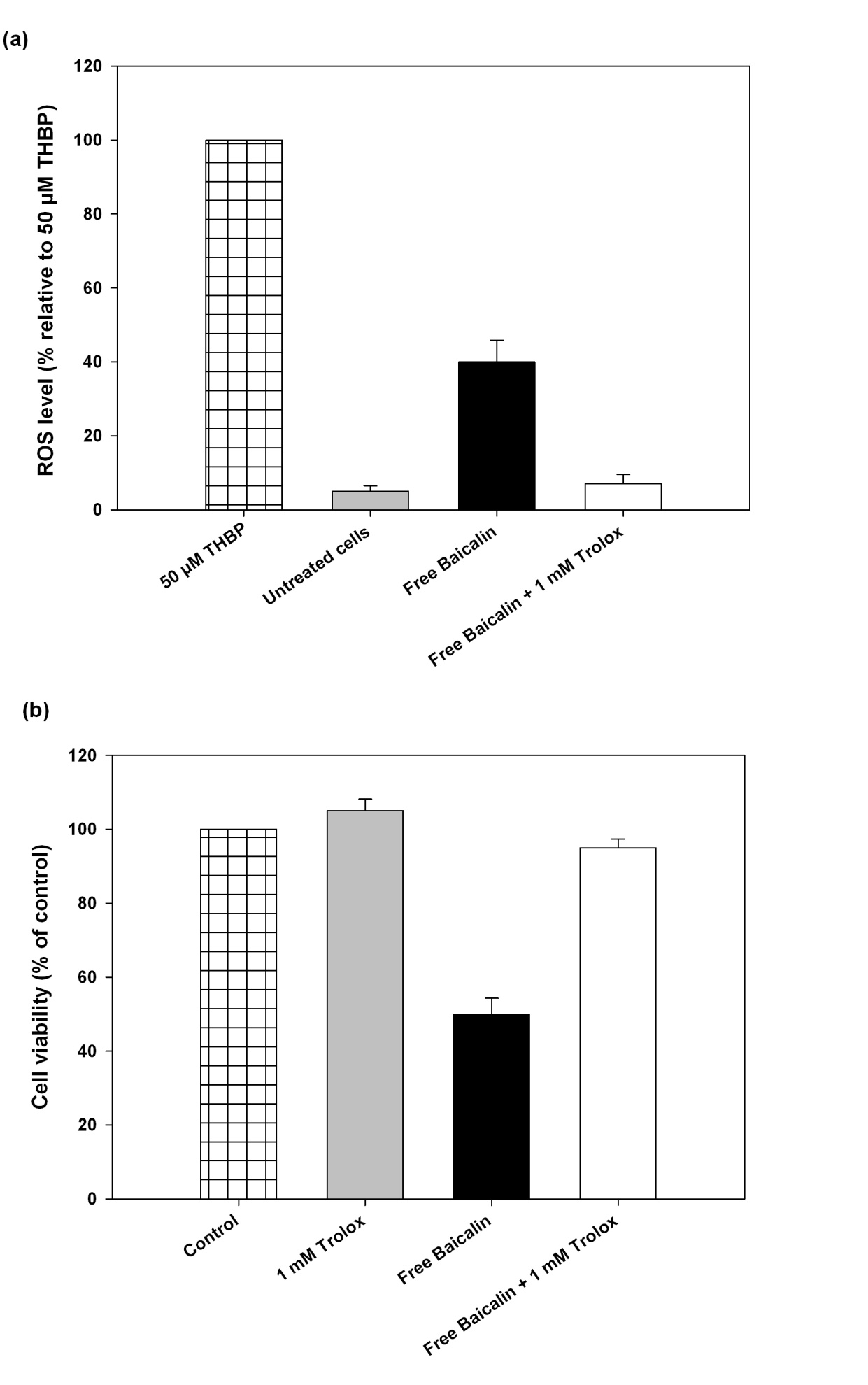

Supplement: Supplementary file 1 — Supplementary file [file 41598_2019_47586_MOESM1_ESM.docx]
